# Prevalence and molecular characterization of *Giardia intestinalis* in racehorses from the Sichuan province of southwestern China

**DOI:** 10.1371/journal.pone.0189728

**Published:** 2017-12-20

**Authors:** Lei Deng, Wei Li, Zhijun Zhong, Xuehan Liu, Yijun Chai, Xue Luo, Yuan Song, Wuyou Wang, Chao Gong, Xiangming Huang, Yanchun Hu, Hualin Fu, Min He, Ya Wang, Yue Zhang, Kongju Wu, Suizhong Cao, Guangneng Peng

**Affiliations:** 1 The Key Laboratory of Animal Disease and Human Health of Sichuan Province, College of Veterinary Medicine, Sichuan Agricultural University, Chengdu, Sichuan Province, China; 2 College of Animal Science and Veterinary Medicine, Henan Institute of Science and Technology, Xinxiang, Henan Province, China; 3 Chengdu Giant Panda Breeding Research Base, Chengdu, Sichuan Province, China; Aga Khan University—Kenya, KENYA

## Abstract

*Giardia intestinalis*, a cosmopolitan zoonotic parasite, is one of the most common causes of protozoal diarrhea in both humans and animals worldwide. Although *G*. *intestinalis* has been detected in many animals, information regarding its prevalence and genotype in Chinese racehorses is scarce. In the present study, we investigated the prevalence of *G*. *intestinalis* in racehorses and performed molecular characterization of the pathogen to assess its zoonotic potential. Two hundred and sixty-four racehorse fecal samples from six equestrian clubs located in different regions of the Sichuan province of southwestern China were examined. Nested polymerase chain reaction (PCR) analysis of the gene encoding triose-phosphate isomerase (*tpi*) showed the prevalence of *G*. *intestinalis* to be 8.3% (22/264), and the prevalence in different clubs varied from 3.6% to 13.5%. Three assemblages were identified in the successfully sequenced samples, including the potentially zoonotic assemblages A (n = 5) and B (n = 14), the mouse-specific assemblage G (n = 3), and a mixed A and B assemblage. Sequence analysis of *tpi*, glutamate dehydrogenase (*gdh*), and beta giardin (*bg*) loci revealed that the majority of sequences isolated from assemblage A were identical to the subtype AIV and assemblage B isolates showed variability among the nucleotide sequences of the subtype BIV. Using the nomenclature for the multilocus genotype (MLG) model, one each of multilocus genotypes A (MLG1) and B (MLG2) were identified, with MLG2 being a novel genotype. To the best of our knowledge, this is the first study to investigate *G*. *intestinalis* in Chinese racehorses. The presence of both animal and human assemblages of *G*. *intestinalis* in racehorses indicated that these animals might constitute a potential zoonotic risk to human beings.

## Introduction

*Giardia intestinalis* (syn. *G*. *lamblia*, *G*. *duodenalis*) is a common flagellate intestinal parasite that can infect a wide range of animals, including humans, domestic animals, and wildlife [1]. So far, at least eight assemblages or genotypes (A to H) of *G*. *intestinalis* have been described based on molecular analysis of *G*. *intestinalis* isolates from different host species [2, 3]. Among them, assemblages A and B, which are considered to be potentially zoonotic, are responsible for the majority of human/ mammalian infections, whereas the other assemblages are host-specific [4]. Generally, assemblages C and D are identified in dogs and are occasionally reported in humans [5]. Assemblage E predominantly infects ruminants and pigs, assemblage F infects cats, assemblage G infects mice and rats, and assemblage H infects marine mammals [6–8].

Giardiasis is transmitted mainly through ingestion of food or water contaminated with *Giardia* cysts [9]. Domestic animals (including race-horses) who frequently feed in pastures irrigated with contaminated water or drink water from contaminated sources may became infected with human Giardia. The clinical symptoms of giardiasis are quite variable, and range from acute or chronic diarrhea to a complete absence of symptoms [10]. *Giardia* species could cause dehydration, abdominal pain, weight loss, and mal-absorption in children or young animals; it has also been reported to cause no or mild symptoms in healthy individuals [11]. Every year, approximately 2.8 × 10^8^ cases of human giardiasis are reported worldwide, and the prevalence rates are 8–30% in developing countries [4, 12]. The World Health Organization (WHO), therefore, classified giardiasis as a neglected tropical disease in 2004 [11].

*Giardia* was first reported in South African horses [13]; since then, this parasite has been identified in horses in other countries, including China [14]. Although *Giardia* causes diarrhea in horses [15, 16], most infected horses do not show any clinical signs, and subclinical effects have also been reported [17]. Assemblages A, B, and E of *G*. *intestinalis* have been detected in horses [17], suggesting that horses might be a potential reservoir of *G*. *intestinalis* that can infect humans or other animals. However, there has been only one study on *G*. *intestinalis* in grazing horses in Xinjiang, China, and very little is known about its prevalence in Chinese racehorses. Therefore, the present study aimed to investigate the prevalence and assemblages of *G*. *intestinalis* in Chinese racehorses to assess their potential for zoonotic transmission.

## Materials and methods

### Ethics statement

The present study protocol was reviewed and approved by the Research Ethics Committee and the Animal Ethical Committee of the Sichuan Agricultural University. All fecal specimens were collected from animals with the permission of the club owners.

### Sample collection

Two hundred and sixty-four fecal samples were collected from racehorses of six equestrian clubs located in different regions of the Sichuan province in southwestern China, between June 2016 and March 2017 (Table 1). There are about 911,000 horses in Sichuan province, and the racehorses have more contact with humans due to they are mainly used for horseback riding, racing and show jumping. Clubs were selected based on the owners’ willingness to participate and the accessibility of animals for sampling. Each racehorse was raised alone in a barn, and the fecal sample was collected separately with sterile gloves after defecation onto the ground, placed into ice-boxes, and transported to the laboratory immediately.

**Table 1:** Prevalence and distribution of *Giardia intestinalis* in different regions of the Sichuan province of southwestern China.

Clubs (region ID)	Regions	No. examined	No. positive (%)	Assemblages (No.)
Club1 (A)	Pixian	48	4(8.3%)	A (1), B (3)
Club2 (B)	Qingbaijiang	52	7(13.5%)	A (2), B (5)
Club3 (C)	Shuangliu	56	2(3.6%)	B (2)
Club4 (D)	Longquan	16	1(6.3%)	B (1)
Club5 (E)	Wenjiang	58	6(10.3%)	A (2), B (1), G (3)
Club6 (F)	Dujiangyan	34	2(5.9%)	B (2)
Total		264	22(8.3%)	A (5), B (14), G(3)

### DNA extraction and PCR amplification

Genomic DNA was extracted directly from each fecal sample using the Stool DNA kit (OMEGA, Norcross, GA, USA) according to the manufacturer’s instructions. The genomic DNA was stored at -20°C until polymerase chain reaction (PCR) amplification. The prevalence and assemblages of *G*. *intestinalis* were determined using nested PCR amplification of the gene encoding triose-phosphate isomerase (*tpi*). Furthermore, *tpi*-positive specimens were analyzed by PCR amplification of the genes encoding beta giardin (*bg*) and glutamate dehydrogenase (*gdh*) [18–20]. The primers and annealing temperatures for the three genes are listed in Table 2. TaKaRa Taq™ DNA polymerase (TaKaRa Bio, Otsu, Japan) was used for all PCR amplifications. Positive and negative controls were included in all PCR tests. All secondary PCR products were subjected to electrophoresis on a 1% agarose gel with ethidium bromide.

**Table 2:** Primer sequences and annealing temperatures of the genes used in this study, as well as the fragment lengths of the PCR products.

Gene	Primer	Sequence (5'-3')	Annealing temperature (°C)	Fragment length (bp)	Reference
*tpi*	F1	AAATIATGCCTGCTCGTCG	50	530	18
	R1	CAAACCTTITCCGCAAACC			
	F2	CCCTTCATCGGIGGTAACTT	50		
	R2	GTGGCCACCACICCCGTGCC			
*bg*	F1	AAGCCCGACGACCTCACCCGCAGTGC	65	530	19
	R1	GAGGCCGCCCTGGATCTTCGAGACGAC			
	F2	GAGGCCGCCCTGGATCTTCGAGACGAC	65		
	R2	CATAACGACGCCATCGCGGCTCTCAGGAA			
*gdh*	F1	TTCCGTRTYCAGTACAACTC	50	511	20
	R1	ACCTCGTTCTGRGTGGCGCA			
	F2	ATGACYGAGCTYCAGAGGCACGT	50		
	R2	GTGGCGCARGGCATGATGCA			

### DNA sequence analyses

The secondary PCR products of the expected sizes were directly sequenced by Life Technologies (Guangzhou, China) using a BigDye® Terminator v3.1 cycle sequencing kit (Applied Biosystems, Carlsbad, CA, USA). Bidirectional sequencing was performed to confirm the accuracy of these sequences. To identify the assemblages and subtypes, the nucleotide sequences obtained in the present study were aligned with the *G*. *intestinalis* reference nucleotide sequences from GenBank and analyzed using BLAST (http://www.ncbi.nlm.nih.gov/BLAST/) and Clustal X 1.83.

### Statistical analysis

The relationship between the prevalence of *G*. *intestinalis*-infected horses and different clubs was analyzed using the chi-square test of the SPSS version 22.0 software (SPSS Inc., Chicago, IL, USA). Differences were considered significant when P < 0.05.

### Nucleotide sequence accession numbers

All nucleotide sequences were submitted to the National Center for Biotechnology Information (NCBI) GenBank database under the following accession numbers: MF169200-MF169206 for *tpi*, MF169193-MF169196 for *bg*, and MF169197-MF169199 for *gdh*.

## Results

### Prevalence and distribution of the assemblages of *G*. *intestinalis* in different horse clubs

The total prevalence of *G*. *intestinalis* was 8.3% (22/264). All the tested clubs were *Giardia*-positive, and the infection rate ranged from 3.6% to 13.5% for the different clubs. The highest prevalence was in Club 2 (7/52, 13.5%), followed by Club 5 (6/58, 10.3%), Club 1 (4/48, 8.3%), Club 4 (1/16, 6.3%), Club 6 (2/34, 5.9%), and Club 3 (2/56, 3.6%) (Table 1).

Among all clubs, 63.6% (14/22) of the *G*. *intestinalis*-positive samples were infected with assemblage B, whereas 22.7% (5/22) were infected with assemblage A and 13.6% (3/22) were infected with assemblage G. Assemblage B was the most common genotype identified in all tested clubs; assemblage A was found in three of six clubs (Clubs 1, 2, and 5), and assemblage G was found only in Club 5 (Table 1).

### Molecular characterization and polymorphisms in *G*. *intestinalis* amplified products

All 22 *G*. *intestinalis*-positive specimens were detected based on three loci, *tpi*, *bg*, and *gdh*. Twenty-two *tpi*, 10 *bg*, and 7 *gdh* sequences were obtained, and four samples were sequenced for all three genes (Table 3). Three *G*. *intestinalis* assemblages (A, B, and G) were revealed for the *tpi* locus; 14 were identified as assemblage B (three different sequences), 5 as assemblage A (two different sequences), and 3 as assemblage G (two different sequences). Of the 5 assemblage A *tpi* nucleotide sequences, three (A7, B34, and B46) were identical to the sequence of KP780964 and the sub-assemblage was AIV (prairie dog from USA); the other two assemblage A (AV) nucleotide sequences (E29 and E34) were identical and showed 100% similarity (509/509 base pairs) with the reference sequence (GenBank accession number KP780973). Of the 14 assemblage B *tpi* nucleotide sequences, genetic polymorphisms were observed at two nucleotide sites (positions 276 and 371) using KM926534 as a reference sequence (Table 4). In the 3 assemblage G *tpi* nucleotide sequences, four base variations were noted at three nucleotide sites compared to the nucleotide sequence JX571041 of a Spanish rodent (positions 165, 228, and 277) (Table 4).

**Table 3:** Assemblages of *Giardia intestinalis* and the distribution of *tpi*, *bg*, and *gdh* sequences for each positive racehorse and multilocus characterization.

Horse ID	*tpi* (subtype)	*bg* (subtype)	*gdh* (subtype)	MLGs
A7	Assemblage A (IV)	Assemblage A (IV-novel-1)		
A15	Assemblage B (IV)			
A17	Assemblage B (IV)			
A40	Assemblage B (IV-novel-1)	Assemblage A (I-novel-1)	Assemblage B (IV-novel-2)	
B15	Assemblage B (IV-novel-1)		Assemblage B (IV-novel-1)	
B30	Assemblage B (IV)		Assemblage B (IV-novel-1)	
B34	Assemblage A (IV)	Assemblage A	Assemblage A (IV)	MLG1
B39	Assemblage B (IV-novel-2)			
B46	Assemblage A (IV)	Assemblage A	Assemblage A (IV)	MLG1
B48	Assemblage B (IV-novel-1)	Assemblage B	Assemblage B (IV-novel-1)	MLG2
B51	Assemblage B (IV)	Assemblage B		
C11	Assemblage B (IV)	Assemblage B		
C14	Assemblage B (IV-novel-2)			
D5	Assemblage B (IV-novel-2)	Assemblage B		
E26	Assemblage G (novel-1)			
E27	Assemblage G (novel-1)			
E28	Assemblage G (novel-2)			
E29	Assemblage A (V)	Assemblage A		
E34	Assemblage A (V)	Assemblage A		
E42	Assemblage B (IV-novel-1)		Assemblage B (IV-novel-1)	
F9	Assemblage B (IV-novel-1)			
F12	Assemblage B (IV-novel-1)			

**Table 4:** Variations in *tpi*, *gdh* and *bg* nucleotide sequences among the different subtypes in assemblages A, B, and G of *G*. *intestinalis*.

Gene	Subtype (n)	Nucleotide at position			GenBank accession no.
*tpi*		276	371		
	Ref. sequence	G	G		KM926534
	BIV (n = 5)	G	G		MF169201
	BIV-novel-1 (n = 6)	G	A		MF169200
	BIV-novel-2 (n = 3)	A	G		MF169202
		165	228	277	
	Ref. sequence	C	G	G	JX571041
	G-novel-1 (n = 2)	T	T	G	MF169204
	G-novel-2 (n = 1)	C	T	A	MF169205
*bg*		63	293		
	Ref. sequence	A	C		KM190700
	AI-novel-1 (n = 2)	G	T		MF169195
*gdh*		112	298	398	
	Ref. sequence	G	C	A	KP687770
	BIV-novel-1 (n = 4)	G	T	A	MF169197
	BIV-novel-2 (n = 1)	A	T	G	MF169198

Among the 10 *bg* locus sequences, two were assemblage A sequences (n = 6) and one was assemblage B sequence (n = 4). The assemblage A sequences (B34, B46, E29, and E34) were identical to the sequence of KM190700 (from water in Canada), and the other two amplified products (A7 and A40) had two base variations compared to the sequence of KM190700 (Table 4). The assemblage B sequences (B48, B51, C11, and D5) were identical to the reference sequences KT948089 (from children in Ethiopian) and KU504731 (from human in Brazil).

Among the 7 sequences of the *gdh* locus, two (B34 and B46) were identified as assemblage A; these sequences were identical to a reference sequence from a cat in Brazil (EF507600). Two of the other 5 amplified products were identified assemblage B sequences, which differed by two and three bases from the reference sequence KP687770 (from a beaver in Canada) (Table 4).

## Discussion

To our knowledge, this study is the first to confirm the presence of *G*. *intestinalis* in racehorses in China; this was determined to be 8.3%. The prevalence of *G*. *intestinalis* in horses shows a wide country-specific variation (0–35%). The prevalence of this parasite has additionally been reported in horses in Brazil (0.5%), Germany (5.4%), grazing horses of Xinjiang (1.5%) [14, 21, 22] and in foals in Netherlands (11.4%), the USA (13%), Belgium (14.2%), Colombia (17.4%), and the Czech Republic (35%) [17, 23–27]. Previous studies have shown that the prevalence of *G*. *intestinalis* in dairy cattle was affected by climate, with significantly higher prevalence in winter than in other seasons [4, 7]. Other studies have also shown that the prevalence of *G*. *intestinalis* was associated with infection of children and foals [24, 28]. The difference between the prevalence of *G*. *intestinalis* obtained in this study and that in other countries may be affected by many factors, such as the examination method, animal age, sample size, management system, timing of specimen collection, and climate.

Although *G*. *intestinalis* occurs in a variety of hosts worldwide, data regarding molecular characterization of the species of equine origin is limited. In previous studies, *G*. *intestinalis* was detected in horses mainly by microscopy or direct immunofluorescence microscopy (DFA) [21–23, 29–31]; however, light microscopy cannot identify trophozoites or cysts of different species [32]. Specific PCR of the *tpi*, *gdh*, and *bg* genetic loci have been used to characterize and classify the genotypes/assemblages of *Giardia* species [3, 6, 33]. So far, horses from 12 countries have been reported to be infected with *G*. *intestinalis*, and assemblages A and B are the most commonly identified assemblages [14, 17]. Assemblages A and B are mainly responsible for human giardiasis; they have also been found in a wide range of animals in China, including dairy cattle, laboratory macaques, sheep, goat, rabbits, dogs, donkeys, and horses, as well as in raw urban wastewater [34–38]. These results suggested that interspecies transmission of *G*. *intestinalis* might be common in China, and horses might be a source of giardiasis outbreaks.

In the present study, assemblage B was found to be more prevalent than assemblage A. Similar results were reported in a previous study in New York State and Western Australia [39]. In contrast, in another study in Italy, 16 and 11 equine specimens were identified to be of assemblages A and B, respectively, and a study in China identified one horse specimen each to be those of assemblages A and B [14, 26]. Interestingly, a recent study reported a horse sample infected with mixed A and B assemblages in Italy [26], which was consistent with the results of this study. Assemblage G is primarily rodent-specific, and has been identified in rodents in Spain, and in mice and rats in Sweden [40, 41]. For the first time, the present study showed that horses could also harbor this assemblage. Furthermore, four *G*. *intestinalis* isolates were amplified at all three loci, forming two multilocus genotypes (MLG1-2). MLG1 (n = 2) was identified as assemblage A and MLG2 was identified as assemblage B. These results indicated the genetic diversity of *G*. *intestinalis* assemblages A and B in Chinese horses.

## Conclusions

In conclusion, the present study is the first to demonstrate the prevalence (8.3%, 22/264) of *G*. *intestinalis* in racehorses of the Sichuan province of southwestern China. We detected the potentially zoonotic assemblages A and B of *G*. *intestinalis*, and identified the mouse-specific assemblage G in racehorses for the first time. Multilocus sequence analysis revealed genetic diversity in assemblages A and B. These results provide basic data on the molecular characterization of the parasite in this region, and further systematic studies are required to investigate the transmission of giardiasis between humans and animals. Moreover, the presence of both animal and human assemblages of *G*. *intestinalis* in racehorses indicated that racehorses might serve as a potential source of infection for human giardiasis, and effective strategies and measures should be implemented to control its transmission from racehorses to humans.
